# 

**Published:** 1909-10-16

**Authors:** 


### GIFTS AND BEQUESTS.

Mr. Alexander Lamberton, of Glasgow, left by his will £200 to the Glasgow Royal Infirmary, and £100 each to the Glasgow Victoria Infirmary, the Glasgow Western Infirmary, and the Royal Hospital for Sick Children.

Mr. George Davidson Deeping, of Hastings, left, under certain contingencies, £1,000 each to Guy's Hospital and the Victoria Hospital, Southend, for a "Deeping" bed.

Miss Frances Mary Yorke, of York, bequeathed £50 each to the York County Hospital and to the York Dispensary.

### APPOINTMENTS.

Dr. Hofmeister, M.V.O., reappointed Port Medical Officer of Cowes.

Dr. F. E. Wynne, Medical Officer of Health to the Leigh Town Council.

The Cromer Urban District Council has appointed Dr. Colvin Smith as medical officer.

Dr. H. Emlyn Jones, Third Medical Inspector under the Essex Education Committee.

Dr. C. E. Rushton, Medical Officer of Health for the Macclesfield Rural District Council.

Dr. B. A. Percival, Assistant Medical Superintendent, Lunatic Asylum, St. Ann's, Trinidad.

Dr. Henry Robinson, M.A., M.D., Anæsthetist to the Cancer Hospital, in place of Dr. N. W. Bourns, resigned.

The Cromer Urban District Council has appointed Dr. Colvin Smith as Medical Officer.

Professor Jordon Lloyd has been appointed by the Council of Birmingham University to succeed Professor Bennett May as Professor of Surgery.

Dr. Frederick A. Sharpe, D.P.H., M.B. Lond., Assistant Medical Officer in place of Dr. E. H. T. Nash, resigned, to the Derby Education Committee.

The vacancy caused by Professor Robinson's resignation of the Professorship of Anatomy at Birmingham University has been filled by the election of Mr. Peter Thompson, M.D., Ch.B. (Vict.), Professor of Anatomy in King's College, London. Professor Thompson was educated at Owens College, Manchester, where he had a distinguished career.

Dr. J. V. Paterson, reappointed Assistant Ophthalmic Surgeon to the Edinburgh Infirmary for a further period of five years as from December 4, 1909.

Mr. Archibald M'Kendrick, F.R.C.S.Ed., Clinical Assistant at the Edinburgh Royal Infirmary.

Mr. A. E. Cullen, M.R.C.S., L.R.C.P., House Physician to the General Infirmary, Chester.

Dr. A. E. Ireland, D.P.H., Medical Officer of Health, Suva, Fiji, and Government Bacteriologist to the Colony.

Dr. C. W. Stidston, M.D., B.S. (Lond.), M.R.C.S., L.R.C.P., Honorary Assistant Surgeon to the Wolverhampton and Staffordshire General Hospital.

Dr. A. W. G. Woodforde, M.B., B.S. (Lond.), Assistant Medical Officer to Mile End Infirmary, E.

Dr. E. Lister Wright, M.R.C.S., L.R.C.P., Medical Officer of Health to the municipality of Simon's Town, Cape Colony, and Assistant Medical Superintendent, Simon's Town Cottage Hospital.

Dr. E. T. Larkham, Medical Officer for the district of Prestwich.

Dr. T. G. Kelby, Medical Officer of Health for the district of Market Bosworth.

Mr. H. N. Keeling, M.R.C.S., L.R.C.P., Medical Officer for the Barlestone district.

Dr. E. Martin, Medical Officer of Health for Springhead Urban District.

Dr. Wyllie Nicol has been appointed physician to the Wards for Skin Diseases; Dr. J. Goodwin Tomkinson physician for Skin Diseases in the out-patient department; Dr. Roy Young extra dispensary surgeon, vice Dr. A. H. Edwards promoted to full dispensary surgeon, at the Western Infirmary, Glasgow.

Dr. Ernest T. Roberts has been appointed chief medical officer for the inspection of children under the Glasgow School Board. Dr. Roberts has held for the past seventeen years the post of certifying factory surgeon of the district of Keighley.

Mr. W. B. Mercer, M.R.C.S., L.R.C.P., has resigned the post of medical officer of health for Rishworth.

Mr. R. C. Colvin Smith, M.R.C.S., L.R.C.P., has been appointed medical officer of health for Cromer Urban District.

### The Question Box.

**SPECIAL NOTICE.**—Questions of interest affecting the honorary medical staff and hospital administration in all departments are invited. When any point arises we hope our readers will formulate it in a question and so co-operate to make this column of real value and interest. In this way the experience of the best-managed hospitals should be made helpful to the whole hospital world. We shall welcome the contribution of both questions and answers.

**N.B.**—The publication of an answer in this column does not necessarily preclude the publication of subsequent answers to the same question, for a question may involve points which require to be threshed out and fully discussed. Answers, if published, will be paid for at scale rates.

### AN EIGHT HOURS' DAY FOR NURSES.

Question 1. Is the eight hours' day for nurses practicable?

ANSWER: No!

A London View.

By the Hon. Sydney Holland, Chairman of the London and Poplar Hospitals.

You ask if an "eight hours' day" for nurses is practicable. My answer is that it is not, and for the following reasons: At the present moment throughout England nurses do not even all get two hours off every day, a half-day once a week and a whole day once a month or once a fortnight. This is the minimum "off time" which nurses may reasonably demand, and no hospital which gives less can be said to be treating its nurses fairly. The excuse where this is not given already is that the expense would be too great, and more bricks and mortar required for which there is no money. These hours are a great improvement on what existed but a few years ago, and to give them has in many cases necessitated a considerable increase in the hospitals' nursing staffs and expensive additions to the existing nurses' homes. It would be quite impracticable, even if it were advisable—which I will deal with later—to add one-third more nurses to the staff of hospitals throughout the country, and to build further additions for these extra nurses.

Take the London Hospital. To divide the twenty-four hours into three shifts would mean an addition of 125 nurses, or, say, £6,250 a year, and about £20,000 spent on building! This would be done to-morrow if it were necessary for the fair treatment of nurses, but it is not. Where nurses get the off time I have set out above—they get more at the "London"—they are not overworked. It is not a profession for a woman who is not strong, but anyone who sees much of hospital nurses must be struck with their healthy appearance, and, may I add, happy faces.

Probably there is no other work to which the arrangement of "an eight hours' day" is so little adapted. Nursing is not merely mechanical labour, because, with the best nurses at any rate, a human interest pervades it. The degree of strain involved in a nurse's period on duty varies enormously in the experience of each individual nurse. At times it is extremely arduous, at others comparatively light. Nurses need, and deserve, their own free time and a liberal allowance of it, but it is best both for them and for their work that their times on duty, whether by day or night, should be adapted to the ordinary customs of daily life which prevail in this country.

Training ought to be made the best preparation possible for the work to follow, and this brings me to the other side of the question—the patient's point of view. In all these discussions about nurses, one too often misses any allusion to the patient.

If "an eight hours' day" prevailed in hospitals it must also prevail in private nursing. How would patients bear the three changes? The change from night to day nurse is bad and trying enough—fancy three changes! And what about the cost? How many patients could pay £6 6s. per week? How often do we receive a letter from a private nurse saying: "This is a case where two nurses are really required to do justice to the patient, but, unfortunately, the people I am with are in poor circumstances, so we must just manage as best we can." Probably such households would regard trained nurses as a luxury entirely beyond their reach if an idea prevailed that three strangers must be admitted to the household to secure the patient being properly nursed! Such an artificial standard as "an eight hours' day" could not possibly tend to promote the best interests of nurses, or of their patients, nor do I believe that the large majority of nurses would desire an arrangement so ill-adapted to the legitimate claims of their work.

And lastly, are the women available? I do not believe there are anything like enough women desirous of becoming nurses to allow of every hospital adding one-third in number to its nurses.

To sum up "an eight hours' day":—

1. It would be harmful to patients.
2. It would be too expensive to patients.
3. It is not required by nurses.
4. It is impossible for hospitals.

### POOR LAW INFIRMARIES.

Question 1. Should Poor Law infirmaries have a visiting medical staff? If so, on what terms and conditions of attendance?

ANSWER: Indeterminate.

A Provincial View.

By Mr. R. W. Marsden, M.D., M.R.C.S., Medical Officer, Crumpsall Workhouse Infirmary.

Though this is the method of management in vogue at the Manchester Workhouse Hospital, and though I consider that the arrangement works apparently satisfactorily, it can hardly be correct to say that it is the only possible system, and that a visiting medical staff is a necessity.

I take it that in the larger infirmaries such as the one at Crumpsall, a sufficient number of junior resident medical officers to ensure thorough medical attendance and attention for the whole institution are indispensable in any scheme. The question then arises, what form should the administrative head assume? In my opinion either of two methods may be adopted, namely, (1) The appointment of a medical man of experience to act as resident medical superintendent, in a similar manner to the positions at present obtaining in asylums or some large fever hospitals, etc.; or (2) a visiting medical officer. In either case I should say a visiting surgeon will probably be required, or, rather, will be highly desirable, since the necessary qualifications for coping with all the various medical and surgical conditions can hardly be expected to be found in the same individual.

The question, therefore, seems to me to resolve itself into this: Should the chief medical officer be resident or non-resident?

If I may take the Manchester Workhouse Infirmary as a sample, I should say that the provision of special accommodation in close proximity to the hospital, the giving of a salary sufficiently adequate to entice and retain a practitioner of any experience, the fact that as a resident official he must still be subservient to the workhouse master (of course, strictly medical matters are excluded)

would be decided inconveniences to any attempt to introduce the resident system. Moreover, seeing that from the point of view of administration the master, who is the responsible head, is always on the premises, and that as regards medical treatment the attendance of the medical officer when non-resident can be quickly obtained by telephone, I do not think that the resident post has any decided advantages as regards the care of the patients over the non-resident one.

There are, however, several indispensable conditions connected with a non-resident medical staff. Thus the staff should not live too far from the hospital to be out of range of urgent calls at any hour; they must be in telephonic communication, a minimum number of regular visits per week should be arranged, and finally the members of the staff should not be so busy in private practice as to feel the allotted visits an excessive demand on their time.

I think with these provisos that the non-resident visiting staff has no practical disadvantages as compared with the alternative system, but on the contrary possesses advantages in the choice of staff, and in their continuity of service which greatly exceed the resident appointment, unless the latter is placed on such a plane as regards expense and freedom of action and position to attract the best men.

### FIRES IN HOSPITALS.

Mr. W. Kay, a member of the House Committee of the Bolton Infirmary, desires answers to the following questions:—

1. What are the principal directions in hospital fire drill for a hospital of 130 beds which is supplied with the usual appliances?
2. In case the ordinary passages are on fire what are the best means to remove patients from the second and third floors of a hospital?

### THE GRADUATES' MEDICAL SCHOOLS.

DIARY FOR THE WEEK, OCTOBER 18 to 23.

### MEDICAL GRADUATES' COLLEGE AND POLYCLINIC, 22 Chenies Street, W.C.

**Clinique.**

**At 4 p.m.**
**October 18**, Dr. J. H. Sequeira, **Skin.**

**At 5.15 p.m.**
**October 18**, Dr. James Collier, **Functional Paralysis.**

**At 4 p.m.**
**October 19**, Dr. Harry Campbell, **Medical.**

**At 5.15 p.m.**
**October 19**, Dr. Thomas Eden, **Malignant Ovarian Tumours.**

**At 4 p.m.**
**October 20**, Mr. James Cantlie, **Surgical.**

**At 5.15 p.m.**
**October 20**, Dr. W. H. B. Stoddart, **The Prognosis of Functional Nervous Disorders.**

**At 4 p.m.**
**October 21**, Sir Jonathan Hutchinson, **Surgical.**

**At 5.15 p.m.**
**October 21**, Dr. Geo. Carpenter, **Heart Disease in Children.**

**At 4 p.m.**
**October 22**, Dr. Scanes Spicer, **Ear, Nose, and Throat.**

### NATIONAL HOSPITAL FOR THE PARALYSED AND EPILEPTIC, Queen Sq., Bloomsbury, W.C.

**At 3.30 p.m.**
**October 19**, Dr. Taylor, **Paraplegia and Its Treatment.**
**October 22**, Dr. Tooth, **Cerebral Tumours.**

### THE POST-GRADUATE COLLEGE, West London Hospital, Hammersmith, W.

**At 10 a.m.**
**October 18, 21**, Surgical Registrar, **Demonstration.**
**October 22**, Medical Registrar, **Demonstration.**

**At 12 noon.**
**October 21**, Dr. Bernstein, **Pathological Demonstration.**

**At 12.15 p.m.**
**October 19**, Dr. Pritchard, **Practical Medicine.**
**October 20, 23**, Dr. Grainger Stewart, **Practical Medicine.**

**At 5 p.m.**
**October 18**, Mr. Pardoe, **Undue Mobility of the Kidneys and Its Consequences.**
**October 19**, Dr. Saunders, **Examination of the Gastric Contents.**
**October 20**, Dr. Beddard, **Medicine. II.**
**October 21**, Dr. Grainger Stewart, **Injuries to Nerves.**
**October 22**, Dr. Abraham, **Cases of Skin Disease.**

The rate of annual subscriptions to The Hospital, either direct from the Office or through agents, is :—For the United Kingdom, 15s. a year; for the Colonies and Abroad, 17s. a year; for the United Kingdom (with Insurance Policy), £1 a year, inclusive of postage in each case.

